# A Novel Approach for Estimating Ovarian Cancer Tissue Heterogeneity through the Application of Image Processing Techniques and Artificial Intelligence

**DOI:** 10.3390/cancers15041058

**Published:** 2023-02-07

**Authors:** Dimitrios A. Binas, Petros Tzanakakis, Theodore L. Economopoulos, Marianna Konidari, Charis Bourgioti, Lia Angela Moulopoulos, George K. Matsopoulos

**Affiliations:** 1 School of Electrical and Computer Engineering, National Technical University of Athens, 15780 Athens, Greece; 2 Department of Radiology, School of Medicine National and Kapodistrian University of Athens, Aretaieion Hospital, 11528 Athens, Greece

**Keywords:** ovarian epithelial cancer, radiomics, artificial intelligence classification, quantitative characteristics, tumor heterogeneity, medical image processing

## Abstract

**Simple Summary:**

High-grade ovarian epithelial cancer (HGOEC) is considered to be among the most fatal gynecological cancers, and it is associated with poor response to treatment and adverse prognosis, possibly due to marked intratumoral heterogeneity. The aim of this study is to present a novel technique that can assess intratumoral cellularity based on quantitative features extracted from medical images, denoted as radiomics, advanced image processing and artificial intelligence algorithms, in an attempt to offer biomedical engineers and health professionals a tool for personalized medicine. According to our results, the average accuracy rating of the proposed method in our study population (*n* = 22) was over 85%.

**Abstract:**

Purpose: Tumor heterogeneity may be responsible for poor response to treatment and adverse prognosis in women with HGOEC. The purpose of this study is to propose an automated classification system that allows medical experts to automatically identify intratumoral areas of different cellularity indicative of tumor heterogeneity. Methods: Twenty-two patients underwent dedicated pelvic MRI, and a database of 11,095 images was created. After image processing techniques were applied to align and assess the cancerous regions, two specific imaging series were used to extract quantitative features (radiomics). These features were employed to create, through artificial intelligence, an estimator of the highly cellular intratumoral area as defined by arbitrarily selected apparent diffusion coefficient (ADC) cut-off values (ADC < 0.85 × 10^−3^ mm^2^/s). Results: The average recorded accuracy of the proposed automated classification system was equal to 0.86. Conclusion: The proposed classification system for assessing highly cellular intratumoral areas, based on radiomics, may be used as a tool for assessing tumor heterogeneity.

## 1. Introduction

High-grade ovarian epithelial cancer (HGOEC), also known as high-grade serous ovarian carcinoma, is considered to be a particularly aggressive form of ovarian cancer accounting for 207,252 deaths in 2020, according to [1]. It is the most common type of ovarian cancer and is characterized by rapid growth and early metastasis [2]. It is the third most common gynecological malignancy worldwide [1], and although there was a decreasing trend in the incidence and mortality rates of ovarian cancer globally between 1980 and 2018, a substantial increase in the incidence of ovarian cancer was observed in younger females [3] and accounted for over 70% of deaths among patients with ovarian carcinoma [4].

The presence of intratumoral heterogeneity, or the diversity of cell types and genetic mutations within a single tumor, is considered to play a significant role in the development and progression of cancer [5]. Intratumoral heterogeneity can make it more difficult to diagnose and treat cancer because different cells within the tumor may respond differently to treatment [6]. For example, some cells may be more resistant to chemotherapy or radiation therapy. Additionally, intratumoral heterogeneity can also lead to the development of therapy-resistant subclones, which can ultimately result in tumor recurrence and poor patient outcomes. Overall, understanding intratumoral heterogeneity is crucial for the development of personalized medicine, as it is important that the treatment strategy takes into account the specific genetic mutations and cellular characteristics of a patient’s tumor [5,6].

In patients with HGOEC, magnetic resonance imaging (MRI) may be used to assess the presence of intratumoral heterogeneity, as it can provide useful information to evaluate the imaging characteristics in the different areas of a tumor. MRI scanning can also help the medical doctor to identify areas of high cellularity, cellular atypia, or necrosis, which may be indicative of tumor heterogeneity. Moreover, functional techniques, such as diffusion-weighted imaging (DWI), are a way to obtain information since highly cellular tissues usually exhibit lower apparent diffusion coefficient (ADC) values than healthy tissue because the water diffusion is restricted by the high cellularity and the disorganized architecture of the tumor [7,8].

The assessment of MRI data can be based on radiomics by providing a more comprehensive and objective characterization of the tumor, allowing for a more accurate diagnosis and prognosis, as they express features of medical images as a series of quantitative data [9,10]. Radiomic features extracted from the tumor’s images can be based on texture, shape, size and intensity, and they can be indicative of the tumor’s biological behavior providing information that is not visible during visual inspection. As ovarian tumors exhibit extensive morphological characteristics [11,12], quantification of the imaging information can be potentially used in fully or semi-automated procedures by employing artificial intelligence techniques in decision support systems (DSS).

In the fairly recent past, radiomics-based analysis has been employed in order to correlate ovarian cancer phenotype, primarily from Computed Tomography (CT) imaging data, with specific gene patterns and survival rates. Based on various studies performed by The Cancer Genome Atlas (TCGA) Research Network, the classification of ovarian cancer (CLOVAR) model was proposed, which relies on microarray transcriptomic profiles, which in turn were used as a prognostic algorithm for high-grade serous ovarian cancer (HGSOC) [13]. Moreover, the relationships between features extracted from CT images and CLOVAR subtypes of HGSOC were assessed [14]. Furthermore, several quantitative metrics acquired from CT imaging data were proposed for expressing spatial inter-site imaging heterogeneity in HGSOC [15]. To our knowledge, the assessment of ovarian cancer heterogeneity in MRI data is not a widely explored subject, especially for HGOEC.

The aim of this study is to present a novel classification method for assessing imaging data and depicting highly cellular tissue within the cancerous tumor based on radiomic features extracted from multiple magnetic resonance imaging sequences. The proposed system identifies intratumoral areas with low ADC values based on the hypothesis that marked tumor heterogeneity may potentially affect prognosis and treatment, as the scope of imaging informatics is to improve efficiency, accuracy and reliability of services among the medical society [16].

## 2. Materials and Methods

### 2.1. Data Acquisition and Datasets

Between March 2019 and March 2022, pelvic MRI scans were performed on 22 women (age: 38–88 years, mean age: 61 years). The data were acquired at the Department of Radiology of the National and Kapodistrian University of Athens in a 3.0-T MRI scanner (Philips Healthcare, Best, The Netherlands). The particular individuals were considered potential HGOEC cases, according to clinical examination and laboratory findings. At the time, none of them had received treatment for HGOEC or had any other conditions requiring an MRI scan.

MR images created a database of 11,095 images with pelvic cancerous regions, with a size of 448 × 448 pixels. According to the acquisition protocol designed for this study, those images came from the following sequences:

- T2-weighted DIXON sequences in the axial plane;
- Diffusion-weighted imaging (DWI) in the axial plane.

### 2.2. Algorithm

The goal of this study is to locate the regions of the tumor with low ADC values. This is achieved through a combination of image processing techniques and artificial intelligence. The overall procedure can be summarized in the following steps, which are performed sequentially:

- Image registration: The process of aligning two or more medical images of the same or different modalities (such as CT, MRI, PET, SPECT, or ultrasound) to analyze them. In our case, the T2-weighted and the DWI images are aligned.
- Image segmentation: The process of dividing an image into multiple regions or segments, each of which corresponds to a different anatomical structure or tissue type. The goal of medical image segmentation is to detect the outline of the cancerous region.
- Image extraction: The process of extracting the cancerous region. The image extraction is needed for both the machine learning pipeline itself and the validation of the whole model through the comparison with the ADC values derived from the DWI sequence.
- Feature extraction: The process of extracting quantitative characteristics, called radiomic features, from the above-mentioned extracted images.
- Feature selection: The process of identifying the subset of the most relevant and useful features from the extracted radiomic features.
- Classification: A machine learning technique that involves training a model to assign a class label to the input data, namely the set of T2-weighted images, on a pixel-by-pixel basis, in an attempt to characterize each region of the data as potentially dangerous or non-dangerous.

**Figure 1:**
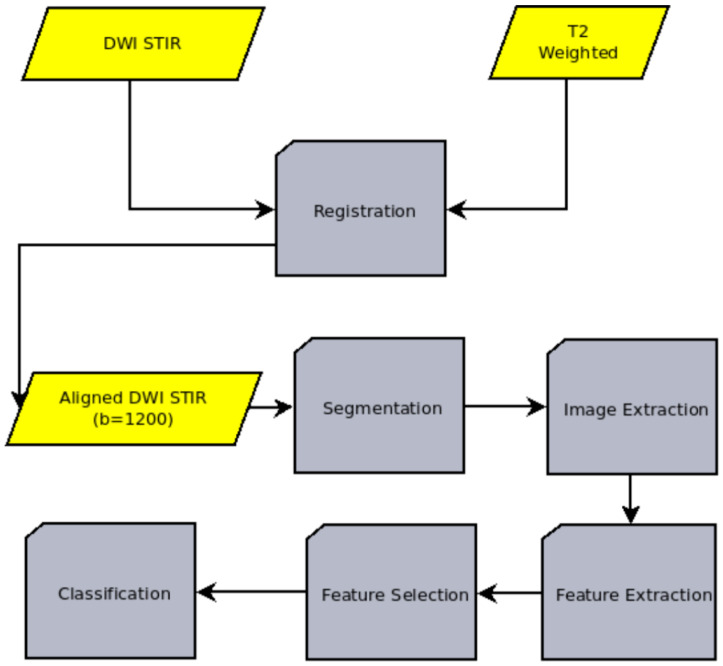
Flow Diagram of the Proposed System.

The overall processing flow of the proposed system is presented in Figure 1.

As can be seen in Figure 1, the first step is to align the images. In order to accomplish this, a two-stage registration technique is employed. Image registration is the spatial alignment of an imaging dataset (moving) with respect to a reference dataset. This is achieved through a recursive process of gradually transforming the images until they are finally aligned. In each iteration, the parameters of a geometrical transformation are calculated according to an optimization algorithm, and the similarity of the transformed images is assessed using a measure of the match [17]. In the case of this study, an initial, fast registration was performed using the Affine transformation, Downhill Simplex as an optimization method and the Mattes mutual information as a measure of the match [18,19]. Subsequently, a second, thorough registration step was performed based on the non-rigid Demon algorithm. The particular technique freely deforms the images by defining an elastic deformation field [20]. The DWI datasets used in this study comprise several subseries corresponding to the different b-value in each case. Those subseries have no spatial differences. Therefore, by applying the above registration scheme, subseries for all b-values are automatically aligned to the T2-weighted images. The same method is employed for aligning the ADC images to the T2-weighted images. ADC image alignment is necessary as ADC maps are used as “the golden standard” to validate the prediction of the model. The accuracy of the alignment is measured quantitatively using the root mean square difference between the point intensities of the initial images that need to be aligned (DWI and ADC datasets) and the reference dataset, which in our case is the T2-weighted dataset [21]. In effect, the smaller this difference is, the better the compared datasets match and hence the more accurate their alignment.

Once both the DWI and ADC image series are spatially matched, it is possible to detect the anatomical region of the tumor from the DWI series. For this purpose, the K-Means clustering algorithm is applied to the images acquired with a b value equal to 1200 s/mm^2^ for automatically identifying the whole tumor [22,23]. Two radiologists with more than 10 years of experience in gynecological imaging were assigned to separately read the original images and select the cancerous regions by manual segmentation in order to validate qualitatively the regions that were automatically extracted through the K-Means clustering approach.

After distinguishing tumorous and non-tumorous areas, it is possible to define the outline of the cancerous region. In each case, the particular region was extracted as a new image. At the same time, the low ADC region of the tumor was automatically extracted from the images derived from the MRI and used as a “golden standard” to validate the proposed model. The ADC cut-off selected to define the “highly cellular tumorous area” was 0.85 × 10^−3^ mm^2^/s according to the radiologists’ personal experience and data obtained from the literature [24,25,26].

In order to assess the heterogeneity of the neoplasm itself, in search of the low ADC region within the tumor, a feature extraction technique was employed to extract the quantitative characteristics of the region of interest. The pre-processed image series, along with their corresponding masks, that is, pixels with useful information versus background, were used, and feature extraction was performed in each slice of the volumetric imaging data, providing a total of 1242 features. After removing the constant features, which are the features that do not differ from image to image, an optimal subset of the original feature vector is selected through a recursive feature elimination cross-validation process [27]. In the case at hand, the aim was to reduce the dimensionality *n* of the feature space *F* in order to overcome the risk of overfitting by recursively evaluating our classification process. To produce a feature ranking, we tested two optimization methods. First of all, a support vector machine (SVM) was used as a type of supervised learning algorithm for the classification of the pixels. The learning method used by SVM is based on the concept of maximal margin classification, which involves finding the hyperplane with the largest possible margin (the distance between the hyperplane and the nearest examples) that separates the classes. This can be formulated as an optimization problem, where the goal is to find the hyperplane that maximally separates the classes while minimizing the number of misclassified examples. To solve this optimization problem, SVMs use the concept of kernel functions, which map the input data into a higher-dimensional space, where it may be easier to find a hyperplane that separates the classes. The kernel function is chosen based on the characteristics of the input data, and it can be used to transform the data into a space where it is linearly separable, even if it is not linearly separable in the original space.

The second method employed during training was stochastic gradient descent (SGD), where the goal is to learn the parameters of the model that minimize the loss function, which measures the difference between the model’s predictions and the true class labels of the examples in the training set. SGD involves iteratively updating the model parameters in a direction that minimizes the loss function. At each step, SGD uses a randomly chosen subset of the training examples (called a mini-batch) to compute an estimate of the gradient of the loss function with respect to the model parameters. The model parameters are then updated in the opposite direction of the gradient, with the step size determined by a learning rate hyperparameter.

Ten-fold cross-validation was implemented in order to minimize the risk of overfitting and the limited data sample [28]. Ten-fold cross-validation is a resampling procedure used to evaluate machine learning models on a limited data sample. This is achieved by splitting the whole dataset into ten groups, excluding one each time and keeping the rest nine groups for training the algorithm. The excluded group is used for validation (testing). This process is repeated 10 times, which is equal to the number of groups created. The efficiency of the produced feature subset was assessed with respect to the limited dataset available for the purposes of this study. Finally, in the testing phase, the points of the tumorous region of a new patient were split into blocks of 400 points (pixels), and the points of every block were classified as low-value ADC region or not, while the selected features of each block were fed into the machine learning classifier for testing. The accuracy score is derived by comparing the predicted value of every point classified by the model to the respective ADC value of the DWI sequence.

### 2.3. Radiomics

In addition to the original images themselves, several other qualitative features were used for classifying the tumorous regions. Those features were extracted by applying the following filters [29,30,31,32]:

- Wavelet filter: A wavelet filter is a mathematical tool used, in our case, to analyze and process image data signals. It is based on the concept of wavelets, which are small, localized functions that can be used to represent signals at different scales and resolutions. This filter applies a wavelet filter to the input image and yields the decompositions and the approximation.
- Laplacian of Gaussian filter: The particular filter applies a Laplacian of Gaussian filter to the input image and yields a derived image for each sigma value specified. A Laplacian of Gaussian image is obtained by convolving the image with the second derivative (Laplacian) of a Gaussian kernel.
- Square filter: Takes the square of the image intensities and linearly scales them back to the original range. It is used to enhance certain features or attributes of the T2-weighted image, such as sharpness and contrast.
- Square root filter: The square root filter is a type of image processing technique that is used to enhance the contrast and texture of the input image. It takes the square root of the absolute image intensities and scales them back to the original range. The square root filter works by raising each pixel value in the image to the power of 0.5 and has the effect of enhancing the contrast of the image, increasing the visibility of small structures.
- Logarithm filter: The logarithm filter works by applying a logarithmic transformation to the pixel intensity values in the image. This transformation maps the pixel values to a new scale, which can help to stretch out the differences between pixels with relatively small intensity values and those with relatively large intensity values. This can make it easier to distinguish between different tissue types or structures within the image.
- Exponential filter: The exponential filter considers the exponential absolute intensity. It works by applying a weighted average to the pixel values in a local region around a central pixel, with the weighting decreasing exponentially as the distance from the central pixel increases.
- Gradient: The gradient returns the magnitude of the local gradient. The gradient is a measure of the local intensity change or slope of an image. It is calculated by taking the derivative of the intensity values in the image with respect to position. The gradient can provide information about the edges and boundaries of objects in an image, as well as the texture and shape of these objects.
- Local Binary Pattern: This is a simple and efficient method for extracting texture features from images. It works by dividing an image into small cells or neighborhoods and comparing the intensity values of each pixel to the center pixel value. Based on the comparison, the pixel is assigned a binary value (0 or 1). These binary values are then concatenated to form a binary pattern, which is a unique descriptor of the texture in the neighborhood.

For the above-mentioned T2-weighted images, the following categories of features were extracted [33]:

- Nineteen features from first-order statistics. First-order statistics are a type of feature that can be extracted from medical images using radiomics techniques. These features describe the overall distribution of intensity values within an image and can provide information about the shape, symmetry and intensity range of the structures in the image. Examples of first-order statistics include the mean, median and standard deviation of the intensity values within an image.
- Shape-based (2D) features. Shape-based features in radiomics involve the measurement and characterization of the shape of structures in the images. These features can include measures of size, shape and spatial relationships, such as the volume, surface area and compactness of a structure. These features are independent of the gray level intensity distribution in the ROI.
- Features from the gray-level cooccurrence matrix describe the second-order joint probability function of an image region. One of these features is autocorrelation. Autocorrelation is a quantitative measure of fineness or coarseness of the texture, which expresses the average intensity difference between a center point and the points of the whole region, indicating the spatial rate of change.
- Sixteen features from the gray-level run-length matrix. This matrix corresponds to the continuous image points that have the same intensity value. One example of such a feature is the run percentage (RP). RP measures the coarseness of the texture by taking the ratio of the number of runs and the number of voxels in a region of interest.
- Sixteen features from gray-level size zone that quantifies gray-level zones in an image. An example of these features is large area emphasis (LAE). LAE is a measure of the distribution of large area size zones, with a greater value indicative of larger size zones and more coarse textures.
- Fourteen features from the gray-level dependence matrix (GLDM) that quantifies gray-level dependencies in an image. An example of these features is dependence non-uniformity (DN). DN measures the similarity of dependence throughout the image, with a lower value indicating more homogeneity among dependencies in the image.
- Five features from the neighboring gray-tone difference matrix, which quantifies the difference between a gray value and the average gray value of its neighbors within a distance *δ*. An example of these features is coarseness, which has been described above.

### 2.4. Evaluation Criteria

In order to evaluate the performance of the proposed classification systems, four well-established metrics were employed: accuracy, balanced accuracy, sensitivity and specificity [34,35,36]. Accuracy considers the sum of the True Positive and True Negative elements as the numerator and the sum of all the entries of the confusion matrix as the denominator.

In other words, it refers to the percentage of correct predictions made by a classifier out of all the predictions made. It is a common metric used to evaluate the performance of a classifier, and it is calculated by dividing the number of correct predictions by the total number of predictions. The basic element of the metric is every pixel of the tumorous area. Each unit has the same weight, and they contribute equally to the accuracy values. [34].

Balanced accuracy is a metric for evaluating the performance of a classifier, specifically when the classes in the data are imbalanced. It is calculated by taking the average of the recall for each class, where recall is defined as the number of True Positive predictions made by the classifier divided by the total number of instances of the class in the data. It is used because our initial set of data is unbalanced [34].

Sensitivity (or recall) is the fraction of True Positive elements divided by the total number of positively classified units. Sensitivity measures the model’s predictive accuracy for the positive class. Specificity, respectively, is the proportion of the True Negatives correctly identified by a diagnostic test. It suggests how good the test is at identifying normal (negative) conditions. In all cases, the results of the classification system were compared against the ADC-based regions, focusing on the highly cellular tumor area.

All image processing routines were developed using the Insight Toolkit (ITK) [37]. The resulting 3D models used for assessing the extracted tumorous regions were visualized through the Visualization Toolkit (VTK) [38]. The classification pipeline framework was implemented using Python 3.6 [39], and the extraction of the radiomics features was based on the pyradiomics 3.0.1 package [40].

## 3. Results

Data from all 22 MRI datasets were processed to produce respective 3D models of the tumors in each case. Initially, the estimated 3D models were assessed qualitatively by means of visual inspection of the extracted tumors. After the extraction of the tumorous regions from the 3D models and the generation of the training data, the proposed classification system was applied to the extracted regions of interest in order to classify their pixels. The validation was performed using the respective ADC values per point derived from the diffusion-weighted images.

Table 1 illustrates the accuracy, balanced accuracy, sensitivity and specificity scores for 22 models, leaving one patient out each time, treated as a new patient. As can be seen in Table 1, the average recorded accuracy of the SGD classification algorithm over all examined cases was 0.89, while the balanced accuracy was estimated to be 0.84.

**Figure 2:**
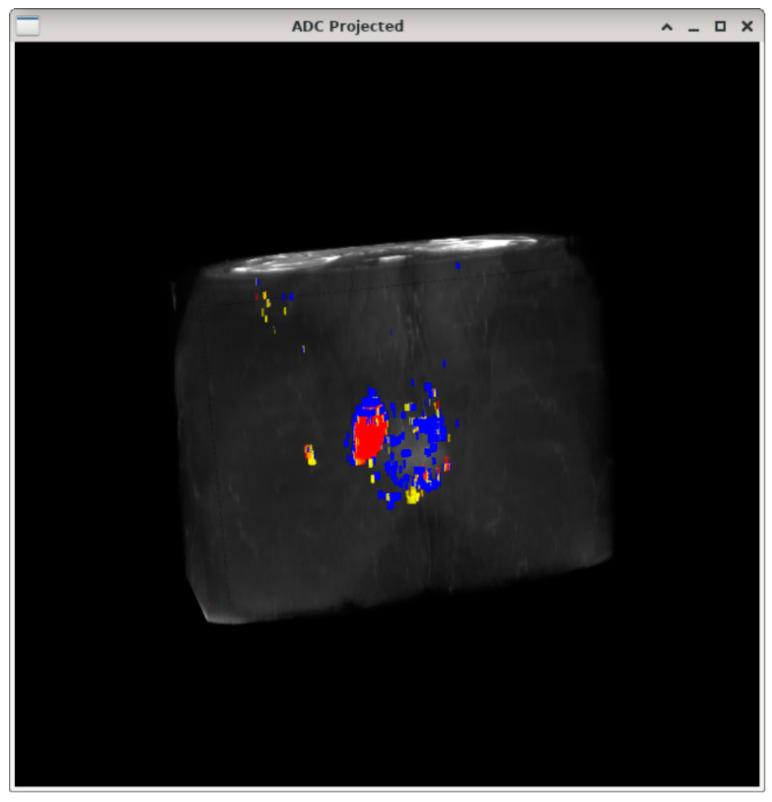
ADC values derived image fused on the T2-weighted sequence. The red region corresponds to the highly cellular tumor area, the blue region corresponds to the low cellular tumor area and the yellow region corresponds to the uncertain tumor area.

**Figure 3:**
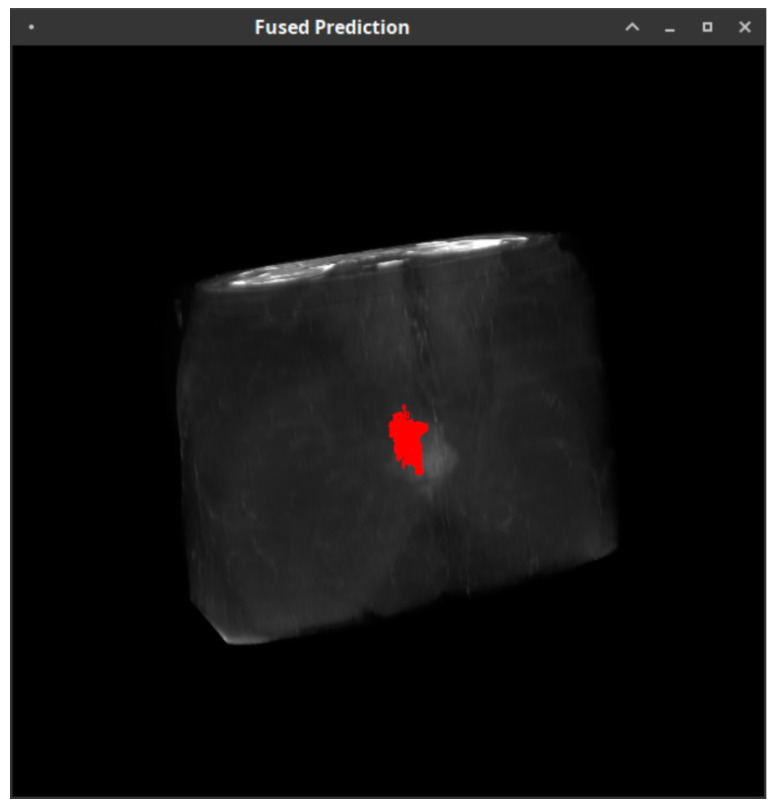
Classification results from the pipeline for the same patient in Figure 2.

Figure 2 depicts the reconstructed 3D volume of the T2-weighted dataset with the tumorous regions superimposed, as these were calculated from the ADC values derived from the DWI sequence using the specified threshold. In the particular example, three heterogeneity regions are depicted, namely, (a) the red region corresponding to the highly cellular tumor area, (b) the blue region, corresponding to the low cellular tumor area and (c) the yellow region, corresponding to the uncertain tumor area, while Figure 3 depicts the highly cellular tumor area that was estimated using the proposed classification pipeline for the same patient, superimposed on the T2-weighted dataset. As can be seen in Figure 2 and Figure 3, the red areas, which correspond to the potentially dangerous cancerous tissue, are derived from the “golden standard”, and the prediction model, respectively, and they match closely, indicating that the proposed classification system performed well for the particular case.

On average, the classification processing pipeline required about 10 min for feature extraction and classification of each patient (depending on the processed datasets). All tests were performed on a common reference system (Intel Core i7-1165G7at 3.5 GHz, 40 GB of RAM, running on Debian 11 GNU/Linux).

## 4. Discussion

The first step of the proposed study was to form a database of MRI imaging data with images from patients suspicious of HGOEC. Those images were used to extract radiomic features. Radiomic features enable us to quantify image characteristics and create a self-learning model for differentiating high and low cellularity areas. The alignment and segmentation techniques applied to develop the model were evaluated quantitatively and qualitatively, while the whole artificial intelligence classification system was tested for possible overfitting over the entire available dataset from the patients recruited during the study.

The presented quantitative and qualitative results demonstrate that the proposed system provides a good prediction performance concerning the highly cellular tumor area. By examining Table 1, it can be concluded that, apart from three patients with low accuracy scores, the majority of the patients showed an accuracy score over 88%, which is considered acceptable regarding the low number of patients used for training. We believe that the limitation of the small number of patients from whom the image database was created leads to a lower accuracy score as the classification accuracy of a machine learning model is generally related to the amount of data that the model has been trained on. In general, as the amount of training data increases, the model’s ability to learn and generalize to new examples tends to improve. This is because a model trained on a larger dataset has the opportunity to learn more about the underlying patterns and relationships in the data and can, therefore, make more accurate predictions.

However, the risk of a possible biased result due to the small number of patients in our dataset is eliminated through the implementation of the specific ten-fold cross-validation method concerning models based on a limited data sample [28].

Another area for potential future work is to use this algorithm to assess the whole tumor or even to study a correlation between biomarkers automatically derived from imaging data and clinical, genomics and/or proteomics data, especially in highly cellular tumors, as radiomics offer huge opportunities to better capture tumor behavior [41].

Identification of intratumoral genetic heterogeneity, or the presence of genetic variations within a single tumor, may help identify areas of unexpected, more aggressive tumor growth. This is because tumors with a high degree of genetic heterogeneity are more likely to contain subclones or subpopulations of cells that have acquired genetic mutations that promote the growth and spread of cancer [42].

One way that intratumoral genetic heterogeneity can be identified is through the use of genomic sequencing techniques, such as whole-genome sequencing or targeted sequencing of specific genes. These techniques can reveal variations in the DNA sequence of different cells within a tumor, allowing for the identification of subclones or subpopulations of cells with distinct genetic profiles. In addition, the spatial distribution of genetic variations within a tumor can also be analyzed through techniques such as single-cell sequencing and spatial transcriptomics, which can help map the location of genetic variations within the tissue.

The knowledge of the presence of subclones with specific genetic characteristics within a tumor can help guide treatment decisions. For example, if a subclone with a specific genetic mutation is identified, targeted therapies that target that mutation may be more effective in treating that particular area of the tumor [43].

Similar studies could be tested on different cancer subtypes where the system can classify different subtypes of cancer based on imaging, genetic, molecular and clinical characteristics. Therefore, another possible area for the presented model to be tested is on different types of abnormalities or lesions in images such as CT or MRI scans.

Consequently, in summary, while similar classification systems could be applied to various medical data, a good assessment of the tumor through the proposed model offers an opportunity for an intratumoral genetic heterogeneity study and hence the identification of areas with potentially more aggressive tumor growth. The identification of specific genetic mutations or subclones within a tumor can also guide treatment decisions and personalized medicine approaches [43,44,45] (personalized medicine).

## 5. Conclusions

The intuitive application programming interface allows for the fast building of medical image segmentation, visualization of the tumor fused in the T2W sequence, pipelines including data I/O, pre-processing, metrics, a library with state-of-the-art feature extraction model and model utilization such as training, tumor area classification as well as fully automatic evaluation.

We propose an effective classification system for assessing highly cellular tumorous areas with the use of radiomics, extracted through a series of pre-processing steps that consist of registration, segmentation and feature extraction on imaging data. The proposed system may be potentially useful for biomedical engineers as a guide to improve the prediction performance based on classification and could even help medical professionals to classify patients into different risk groups that can lead to personalized medicine. Accurate preoperative classification of HGOEC patients into risk categories may be associated with early customization of treatment, optimizing the therapeutic outcome and thus ultimately improving patients’ survival rates [46].

## Figures and Tables

**Table 1:** Prediction Results.

Patient	Accuracy		Balanced Accuracy		Sensitivity		Specificity	
	SVM	SGD	SVM	SGD	SVM	SGD	SVM	SGD
1	0.55	0.78	0.69	0.55	0.44	0.97	0.94	0.12
2	0.99	1	0.86	1	0.99	1	0.73	1
3	0.99	1	0.91	0.96	0.99	1	0.81	0.92
4	0.98	1	0.99	1	0.98	1	1.00	1
5	0.86	1	0.93	1	0.86	1	1.00	1
6	0.98	1	0.50	1	1.00	1	0.01	1
7	0.63	0.68	0.58	0.77	0.67	0.59	0.49	0.94
8	0.99	0.98	0.99	0.99	0.99	0.98	1.00	1
9	0.92	0.99	0.94	0.99	0.92	0.99	0.96	0.99
10	0.88	0.85	0.77	0.77	0.92	0.88	0.62	0.65
11	0.97	0.97	0.91	0.94	0.98	0.98	0.83	0.9
12	0.87	0.68	0.87	0.83	0.87	0.67	0.87	1
13	0.99	0.99	0.99	0.86	0.99	0.99	1.00	0.73
14	0.80	0.97	0.77	0.86	0.80	0.98	0.75	0.74
15	0.95	0.98	0.95	0.98	0.90	0.97	1.00	0.99
16	0.74	0.65	0.79	0.74	0.69	0.58	0.89	0.89
17	0.81	0.93	0.78	0.58	0.81	0.94	0.75	0.22
18	0.77	0.88	0.77	0.88	0.85	0.79	0.69	0.97
19	0.99	0.99	0.73	0.62	0.99	0.99	0.48	0.25
20	0.92	0.87	0.67	0.78	0.99	0.9	0.36	0.66
21	0.48	0.59	0.71	0.63	0.42	0.57	1.00	0.69
22	0.87	0.79	0.84	0.86	0.87	0.78	0.81	0.93
Average	0.86	0.89	0.81	0.84	0.86	0.89	0.77	0.8
±Std. Dev.	0.14	0.14	0.13	0.15	0.16	0.15	0.25	0.27

## Data Availability

Imaging data were provided by the Department of Radiology of the Aretaieion Hospital. Although these data can be available upon request, they are not uploaded to publicly accessible links due to the General Data Protection Regulation (GDPR) policy of the Hospital.
